# Impact of Self-Acupressure on Co-Occurring Symptoms in Cancer Survivors

**DOI:** 10.1093/jncics/pky064

**Published:** 2019-01-16

**Authors:** Suzanna Maria Zick, Ananda Sen, Afton Luevano Hassett, Andrew Schrepf, Gwen Karilyn Wyatt, Susan Lynn Murphy, John Todd Arnedt, Richard Edmund Harris

**Affiliations:** 1 Department of Family Medicine; 2 Department of Nutritional Sciences, University of Michigan, Ann Arbor, MI; 4 Department of Biostatistics, University of Michigan, Ann Arbor, MI; 5 Department of Anesthesiology, University of Michigan, Ann Arbor, MI; 6 College of Nursing, Michigan State University, East Lansing, MI; 7 Physical Medicine and Rehabilitation, University of Michigan, Ann Arbor, MI; 8 Physical Medicine and Rehabilitation, VA Ann Arbor Health Care System, GRECC, Ann Arbor, MI; 9 Sleep and Circadian Research Laboratory; 10 Departments of Psychiatry and Neurology, University of Michigan, Ann Arbor, MI

## Abstract

**Background:**

Cancer survivors with fatigue often experience depressive symptoms, anxiety, and pain. Previously, we reported that self-acupressure improved fatigue; however, its impact on other co-occurring symptoms and their involvement in treatment action has not been explored.

**Methods:**

Changes in depressive symptoms, anxiety, and pain were examined prior to and following two formulas of self-acupressure and usual care using linear mixed models in 288 women from a previously reported clinical trial. Participants were categorized by random assignment into one of three groups: 1) relaxing acupressure, 2) stimulating acupressure, or 3) usual care. Moderators investigated were body mass index, age, depressive symptoms, anxiety, sleep and pain, and mediators were change in these symptoms.

**Results:**

Following treatment, depressive symptoms improved statistically significantly for the relaxing acupressure group (41.5%) compared with stimulating acupressure (25%) and usual care (7.7%). Both acupressure groups were associated with greater improvements in anxiety than usual care, but only relaxing acupressure was associated with greater reductions in pain severity, and only stimulating acupressure was associated with greater reductions in pain interference. There were no statistically significant moderators of sleep quality, anxiety, or depressive symptoms. Fatigue statistically significantly moderated pain, and age statistically significantly modified fatigue. Changes in depressive symptoms and sleep quality statistically significantly mediated the relationship between relaxing acupressure and usual care on fatigue; however, the effect was small.

**Conclusions:**

Acupressure was associated with greater improvements than usual care in anxiety, pain, and symptoms of depression in breast cancer survivors with troublesome fatigue. These findings warrant further evaluation in suitably controlled randomized trials.

Many breast cancer survivors (BCS) experience multiple co-occurring symptoms that persist long after cancer treatments end (1–5). These symptoms include fatigue, chronic pain, anxiety, depression, and poor sleep and affect from 25% to more than 40% of BCS (2,6–8), causing poor quality of life (9,10), difficulty keeping and maintaining employment (11,12), and challenges with friends and families (13,14).

Treatments that address multiple symptoms simultaneously could be of value to BCS. One possible treatment is self-acupressure, a component of Traditional Chinese Medicine (TCM), wherein pressure is applied with the thumb, finger, or device to points throughout the body to improve symptoms. This intervention has been used to treat fatigue (15), poor sleep (16,17), chronic pain (18), depression (19–21), and anxiety (19,21). However, acupressure’s simultaneous effect on multiple symptoms in BCS has yet to be examined.

Previously we confirmed that two distinct acupressure formulas, relaxing acupressure (a TCM formula for improving sleep) and stimulating acupressure (a TCM formula for improving energy), were effective for reducing fatigue (22,23). For sleep quality, only the relaxing acupressure demonstrated improvement (23), suggesting some specificity for acupressure point stimulation. The effects of different forms of acupressure on depressive symptoms, anxiety, or chronic pain in BCS are uncertain. It is also unknown if the presence of one or multiple symptoms changes the likelihood of acupressure relieving co-occurring symptoms (moderators) or if improvements in these symptoms are the mechanism (mediators) through which self-acupressure works.

The purpose of this post hoc exploratory analysis was to examine the differential effect of relaxing and stimulating self-acupressure and usual care on the secondary outcomes of depressive symptoms, anxiety, and chronic pain in fatigued BCS. We also explored if these symptoms in addition to fatigue and poor sleep acted as either moderators or mediators of the two self-acupressure treatments.

## Methods

### Trial Design, Participants, and Interventions

This study is a secondary data analysis addressing unanswered questions from the main study. Parent study methods have been reported previously (23,24). In brief, we performed a 10-week, randomized trial involving two self-administered acupressure protocols, relaxing and stimulating, and usual care. Acupressure was self-administered daily for 6 weeks followed by a 4-week washout period. Participants had five acupressure visits: screening, baseline, 3 week, 6 week (end of treatment), and 10 week (end of washout phase). The following data were collected in person: clinical and sociodemographic data were collected at baseline; depressive and anxiety symptoms, sleep quality, and pain were captured at baseline and weeks 6 and 10; and fatigue was collected weekly from baseline through week 10. These in-person data collection points were comparable in providing attention for all three groups.

Eligible participants were female BCS who had stage 0 to III breast cancer, completed primary cancer treatments 12 months or more prior, and were experiencing persistent fatigue (≥4 on the Brief Fatigue Inventory [BFI] for ≥3 months that started at or after their cancer diagnosis) (25). Women were ineligible if they were planning to start/stop a new treatment for any indication, taking insomnia medications, received acupuncture or acupressure in the last 6 months, had an untreated major depressive disorder, or had other fatigue-causing comorbidities.

Women were taught to self-administer acupressure daily in addition to usual care by stimulating each point in a circular motion for 3 minutes. Participants were taught acupressure by one of 13 acupressure educators who were taught by a certified acupuncturist (24). Relaxing acupressure consisted of: *Yin tang*, *Anmian*, Heart 7, Spleen 6, and Liver 3. All acupoints were performed bilaterally except for *Yin tang*, which was done centrally. Stimulating acupressure points comprised *Du* 20, Conception Vessel 6, Large Intestine 4, Stomach 36, Spleen 6, and Kidney 3. Points were administered bilaterally except for *Du* 20 and Conception Vessel 6, which were performed centrally (Supplementary Figure 1, available online) (26).

Our self-acupressure intervention fidelity was reported previously (23,24,27). Briefly, fidelity of the educators’ delivery was assessed both immediately after being trained by the certified acupuncturist and again after training several participants. Study participants were assessed immediately after being trained by an educator and at the end of acupressure treatment.

This trial is registered at clinicaltrials.gov Identifier NCT01281904 (https://clinicaltrials.gov/ct2/show/NCT01281904). The study was approved by the University of Michigan Medical School, Michigan State University, and Michigan Department of Public Health Institutional Review Boards, and participants provided written informed consent.

### Outcome Measures

To measure fatigue, we used the BFI (25). This scale was validated in cancer patients (Cronbach alpha ≥0.95) (28,29). The BFI assesses severity and impact of fatigue during the past 24 hours. The instrument consists of 9 items, each measuring fatigue on a 0–10 scale, and is calculated from the mean of completed items. Scores of four or more indicate clinically relevant fatigue (25). A three-point reduction is a clinically meaningful improvement and a decrease to four indicates remission (30).

Sleep quality was assessed by the 19-item Pittsburgh Sleep Quality Index (PSQI). It evaluates sleep disturbance during the past month. PSQI yields a global score with a Cronbach alpha of 0.81 (28). A score of five or more suggests poor sleep quality (28). A three-point reduction implies clinically meaningful improvement, and less than five suggests a remission of sleep disturbance (31).

Depressive and anxiety symptoms were measured with the Hospital Anxiety and Depression Scale (HADS), a 14-item questionnaire measuring symptoms in the past week. A meta-analysis of studies using HADS reported a mean Cronbach alpha of 0.83 for anxious symptoms and 0.82 for depressive symptoms (32,33). Scores greater than 10 are considered indicative of a likely case of either depression or anxiety (33).

Clinical pain was measured by a pain visual analog scale (VAS) and the Brief Pain Inventory (BPI). The VAS is a 10-cm line anchored by the words “no pain” and “worst possible pain.” Using a ruler, the score is determined by measuring the distance between the “no pain” anchor and the participant’s mark, providing a range of scores from 0 to 10. The BPI has nine items measuring pain severity (0 = no pain; 10 = pain as bad as you can imagine) and its functional impact (0 = no interference; 10 = interferes completely). Mean values of three or more on either severity or interference are considered clinically significant pain. A 1.1- to 3-point decrease, 20% drop of pain from baseline, or drop below 3 is generally considered clinically meaningful both for the VAS and the BPI (34).

### Statistical Analysis

Analysis of variance and Pearson χ^2^ were used to test balance between groups on baseline characteristics. For all four outcomes (anxiety, depressive symptoms, pain severity, and pain interference), separate linear mixed models were used to investigate time by treatment effects. In the linear mixed models, a random subject intercept was included to account for subject clustering; visit (baseline, end of treatment, washout), group, and the interaction term (visit-by-group), and any variable that was statistically significantly different across groups at baseline (eg, pain medication use [Yes/No]) were included as fixed effects. For pairwise comparisons, a Bonferroni-corrected experiment-wise *P* value of .05 or less (obtained by multiplying the unadjusted *P* values by 3) was considered statistically significant. The associated confidence intervals were also adjusted accordingly. All tests were two-sided.

Moderation analysis was performed using logistic regression to investigate the impact of baseline pain interference, anxiety, depressive symptoms, sleep quality, age, and body mass index on improvement at the end of acupressure treatment. Symptom improvements were defined as the clinical cutoff values for the binary outcome of interest: fatigue (percentage of participants with BFI <4), sleep quality (percentage of participants with PSQI <5), anxiety (percentage of participants with HADS anxiety <8), depression (percentage of participants with HADS depression <8), and pain interference (percentage of participants with BPI <3). In each model, improvement status at the end of acupressure treatment was the outcome, whereas group assignment and the interaction term (group-by-moderator) were the predictor variables. A *P* value of .05 or less for the interaction term was considered statistically significant moderation.

Mediation analyses were conducted using a logistic regression framework (PROCESS macro 2.16.3 for SPSS). The independent variable was group assignment, the dependent variable was fatigue status at end of acupressure treatment, and the mediating variables were change scores in HADS depression, HADS anxiety, BPI interference, and PSQI scores. The significance of indirect effects was tested by using 95% bias-corrected bootstrapped confidence intervals. Both participant age and body mass index were covariates in these models.

For both moderation and mediation analyses, the sample was restricted to participants who were at or above clinical cutoff values for the outcome of interest (eg, depression models were conducted only in participants having ≥8 HADS depression scores at baseline). Also, pain severity was not investigated in these models because pain interference and severity were highly correlated (*r* > 0.70) and pain interference is viewed as a better indicator of function and quality of life.

This study was powered to detect a statistically significant difference through time between treatment arms for fatigue and sleep quality at the end of acupressure, and not specifically powered to detect changes in secondary patient-reported outcomes. Power analysis details are described elsewhere (23).

## Results

### Participant Characteristics

**Figure 1.**
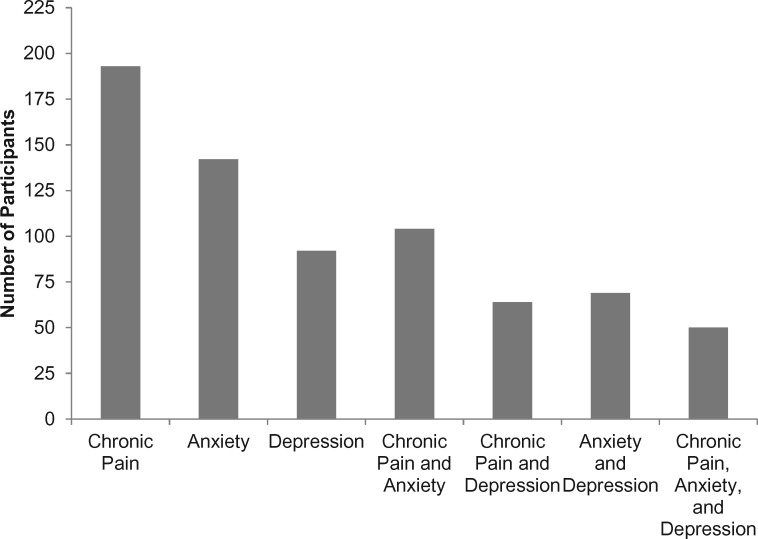
Number of participants at baseline reporting one or more symptoms of chronic pain, depression, or anxiety. Chronic pain was defined as three or more on the Visual Analog Scale at baseline, anxiety was defined as eight or more on the Hospital Anxiety Depression Scale (HADS) at baseline, and depression was defined as eight or more on the HADS Depression Scale at baseline. All participants had persistent fatigue, defined as having a Brief Fatigue Inventory score of four or more at baseline.

Initial screening involved 424 women, and 288 were categorized by random assignment. At baseline 193 reported chronic pain and 92 met criteria for a likely case of depression and 142 for anxiety. Some women had multiple symptoms at baseline, with 50 women reporting all three symptoms (Figure 1). Six weeks of acupressure were completed by 144 of the 193 with chronic pain, and 65 women with depression and 102 with anxiety completed the study. Supplementary Figures 2–4 (available online) document the number of participants categorized by random assignment to each study group, exclusions, and reasons for discontinuing acupressure.

There were no baseline differences between study participants (n = 288) (Table 1). There were also no statistically significant sociodemographic or clinical differences among the three subsets of women with the exception of women with chronic pain in the stimulating acupressure group, who were statistically significantly more likely to be treated with hormone therapies for their cancers (n = 46, 79%, *P* = .036) and to take omega-3 supplements (n = 24, 42%, *P* = .019). Similarly, women with anxiety in the stimulating acupressure group were also statistically significantly more likely to take omega-3 supplements (n = 20, 37%, *P* = .011) (Supplementary Tables 1–3, available online).

**Table 1.** Sociodemographic and clinical characteristics at baseline

Characteristic	All participants (n = 288)	Chronic pain at baseline (n = 193)	Depressed at baseline (n = 92)	Anxious at baseline (n = 142)
Demographics				
Age, mean (SD), y	60.5 (10.0)	60.5 (9.6)	60.2 (8.9)	59.2 (9.3)
Race, n (%)				
White	258 (90)	168 (87)	77 (84)	121 (85)
Clinical characteristics				
BFI, mean (SD)	5.1 (1.5)	5.3 (1.5)	5.6 (1.6)	5.3 (1.6)
BPI, mean (SD)				
Severity	3.8 (2.1)	4.6 (1.8)	4.1 (2.3)	4.1 (2.1)
Interference	3.2 (2.4)	4.0 (2.2)	4.0 (2.5)	3.7 (2.4)
HADS, mean (SD)				
Anxiety	7.8 (4.0)	8.2 (3.9)	10.2 (3.6)	11.0 (2.7)
Depression	6.2 (3.2)	6.3 (3.2)	9.9 (1.8)	7.6 (3.0)
Stage of cancer, n (%)				
Stage 0*	67 (23)	43 (22)	19 (21)	38 (27)
Stage I	88 (31)	64 (33)	34 (37)	45 (32)
Stage II	76 (26)	48 (25)	20 (22)	32 (23)
Stage III	32 (11)	22 (11)	10 (11)	14 (10)
Unknown	22 (8)	14 (7)	8 (9)	11 (8)
Estrogen receptor status				
Yes	191 (66)	129 (67)	59 (64)	90 (63)
No	57 (20)	35 (18)	18 (20)	28 (20)
Unknown	22 (8)	14 (7)	15 (16)	24 (17)
Menopausal status				
Premenopausal	105 (37)	66 (34)	29 (32)	55 (39)
Perimenopausal	22 (8)	16 (8)	10 (11)	13 (9)
Postmenopausal	153 (53)	105 (54)	50 (54)	70 (49)
Unknown	8 (3)	6 (3)	3 (3)	4 (3)
Time since cancer diagnosis, mean (SD), y†	5.5 (3.6)	5.6 (3.9)	5.3 (2.6)	5.3 (3.3)
Treatments received, n (%)‡				
Surgery	287 (100)	192 (100)	91 (99)	141 (99)
Chemotherapy	133 (46)	85 (44)	39 (42)	60 (42)
Radiation	205 (71)	141 (73)	63 (69)	98 (69)
Hormone therapy	192 (67)	127 (66)	61 (66)	89 (63)
Medications (% taking at baseline)				
Acetaminophen	76 (28)	53 (28)	29 (32)	34 (25)
Anticonvulsants	33 (12)	28 (15)	11 (12)	19 (14)
Aromatase inhibitors	66 (24)	44 (24)	19 (21)	31 (23)
Benzodiazepines	28 (10)	22 (12)	14 (16)	23 (17)
Glucosamine/chondroitin	26 (10)	20 (11)	8 (9)	14 (10)
NDRIs	15 (6)	11 (6)	7 (8)	11 (8)
NSAIDs	179 (65)	124 (66)	53 (59)	92 (68)
Omega-3s	85 (31)	59 (32)	28 (31)	35 (26)
Opiates and morphinomimetrics	36 (13)	34 (18)	15 (17)	21 (15)
SERMs	40 (15)	24 (13)	18 (20)	22 (16)
SNRIs	38 (14)	29 (16)	20 (22)	24 (18)
SSRIs	51 (19)	38 (20)	22 (24)	30 (22)
Tricyclics	8 (3)	8 (4)	5 (6)	6 (4)
Other§	26 (10)	21 (11)	10 (11)	15 (11)

* Stage 0 includes ductal carcinoma in situ (DCIS) and Lobular carcinoma in situ (LCIS). BFI = Brief Fatigue Inventory; BPI = Brief Pain Inventory; HADS = Hospital Anxiety and Depression Scale; NDRIs = norepinephrine-dopamine reuptake inhibitors; NSAIDs = nonsteroidal anti-inflammatory drugs; SERMs = selective estrogen receptor modulators; SNRIs = serotonin-norepinephrine reuptake inhibitors; SSRIs = selective serotonin reuptake inhibitors.

† Time since cancer diagnosis was calculated from on-study date and date of diagnosis in years.

‡ Percentages may not add up to 100% because participants can receive multiple treatments or diagnoses.

§ Other medications include trazodone, lisinopril, flexeril, fioricet, imitrex, norgesic, fiorinal, butalbital, remeron, buspirone, milk thistle, ginger biloba, medical marijuana, St. John’s wort, pramipexole, requip, adderall, buspar, tamoxilen, atenolol, omeprazole, baby aspirin, vitamin D, vitamin E, and topical lidocaine.

### Depression

**Figure 2.**
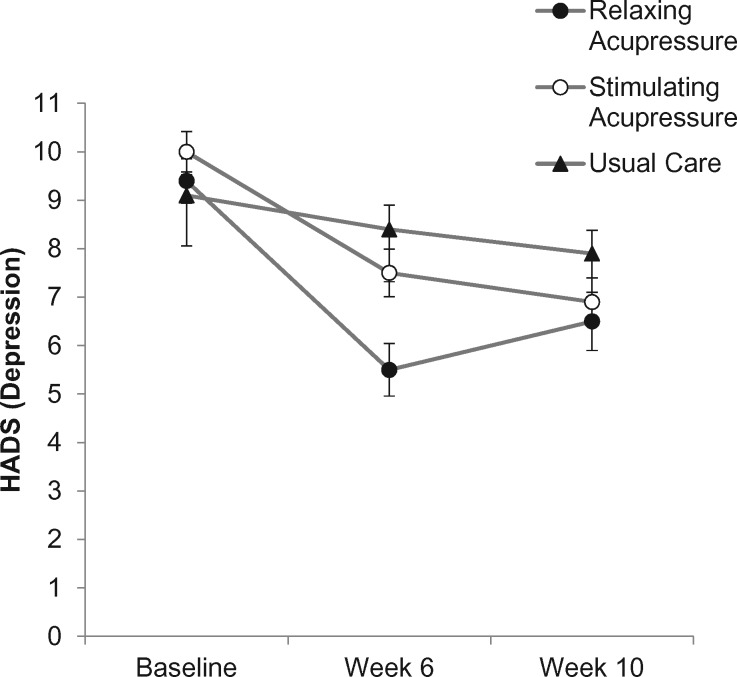
Change in depression across study arms (relaxing acupressure, stimulating acupressure, and usual care) as measured at baseline, 6 weeks after baseline (end of acupressure or usual care treatment), and 10 weeks after baseline (4 weeks post-acupressure or usual care treatment). HADS = Hospital Anxiety and Depression Scale.

Following treatment, relaxing acupressure was associated with statistically significantly better reductions in depressive symptoms than both usual care and stimulating acupressure (the adjusted mean ± 95% CI change in HADS depression subscale, relaxing acupressure vs usual care = −2.83, 95% CI = 4.68 to −1.05, *P* < .001, relaxing acupressure vs stimulating acupressure = −1.95, 95% CI = −3.71 to −0.19, *P* = .025). Stimulating acupressure was not different from usual care (*P* = .63). The percentage improvement in depressive symptoms at end of treatment was 41.5%, 25.0%, and 7.7% for relaxing acupressure, stimulating acupressure, and usual care, respectively. At 10 weeks, there were no statistically significant differences between any groups (Table 2; Figure 2).

**Table 2.** Pain, anxiety, and depression by group assignment and study visit

	Baseline visit			Week 6 visit				Week 10 visit				
	Mean (SD)			Mean (SD)				Mean (SD)				
Variable	Relaxing acupressure	Stimulating acupressure	Usual care	Relaxing acupressure	Stimulating acupressure	Usual care	95% CI‡	Relaxing acupressure	Stimulating acupressure	Usual care		95% CI‡
BPI pain severity*	3.8 (0.35)	3.8 (0.38)	3.9 (0.33)	2.7 (0.35)	2.9 (0.38)	4.3 (0.33)	−1.16 to 0.83§	2.7 (0.35)	3.0 (0.38)		3.9 (0.33)	−1.19 to 0.80§
							−1.84 to 0.09‖					−1.40 to 0.53‖
							−1.98 to −0.10¶					−1.57 to 0.30¶
BPI pain interference*	4.7 (0.31)	4.3 (0.34)	4.5 (0.29)	3.5 (0.31)	3.3 (0.34)	4.6 (0.30)	−0.65 to 1.24§	3.8 (0.31)	3.4 (0.34)		4.5 (0.30)	−0.59 to 1.30§
							−1.98 to −0.15‖					−1.52 to 0.32‖
							−1.66 to 0.13¶					−1.13 to 0.65¶
HADS anxiety†	10.3 (1.13)	10.4 (1.10)	10.4 (1.04)	8.6 (1.15)	8.6 (1.13)	10.5 (1.08)	−1.74 to 1.97§	9.0 (1.17)	8.5 (1.13)		10.4 (1.08)	−1.27 to 2.58§
							−3.66 to −0.21‖					−3.72 to −0.27‖
							−3.60 to −0.04¶					−3.17 to 0.50¶
HADS depression	9.4 (0.46)	10.0 (0.42)	9.1 (0.43)	5.5 (0.54)	7.5 (0.49)	8.4 (0.50)	−3.71 to −0.19§	6.5 (0.60)	6.9 (0.50)		7.9 (0.48)	−2.28 to 1.47§
							−2.56 to 0.81‖					−2.70 to 0.64‖
							−4.60 to −1.05¶					−3.28 to 0.41¶

* Adjusted for omega-3 use, hormone therapy treatment. BPI = Brief Pain Inventory; HADS = Hospital Anxiety and Depression Scale.

† Adjusted for omega-3 use.

‡ Derived from a linear mixed model, bolded statistically significant at *P* less than .05, Bonferroni-corrected for pairwise comparisons.

§ Relaxing acupressure compared with stimulating acupressure.

‖ Stimulating acupressure compared with usual care.

¶ Relaxing acupressure compared with usual care.

### Pain Severity and Interference

**Figure 3.**
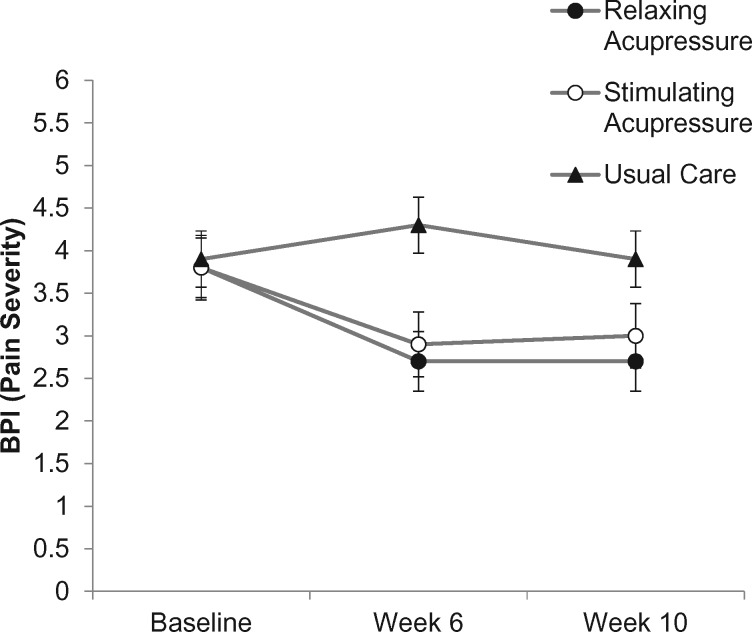
Change in pain severity across study arms (relaxing acupressure, stimulating acupressure, and usual care) as measured at baseline, 6 weeks after baseline (end of acupressure or usual care treatment), and 10 weeks after baseline (4 weeks post-acupressure or usual care treatment). BPI = Brief Pain Inventory.

Following treatment, the adjusted mean ± 95% CI change in BPI severity from baseline was statistically significantly lower in the relaxing (*P* < .001) and stimulating acupressure (*P* = .016) groups. The effect continued to be statistically significant at the 10-week period for the relaxing acupressure arm (*P* = .002) but was no longer statistically significant in the stimulating arm (*P* = .07). The reduction represented a 28.9% and 23.7% improvement in pain severity in the relaxing and stimulating acupressure groups, respectively. No statistically significant changes were observed within the usual care arm. Compared with usual care, mean pain severity was statistically significantly lower in the relaxing acupressure arm (relaxing acupressure: OR = −1.04, 95% CI = −1.98 to −0.10, *P* = .024 vs usual care) following treatment. Although there was a reduction in the stimulating acupressure arm compared to the usual care arm, it was not statistically significant after adjusting for multiple comparisons (stimulating acupressure: OR = −0.88, 95% CI = −1.84 to 0.09, *P* = .09 vs usual care). No statistically significant pairwise difference was observed at the 10-week point (Table 2, Figure 3).

**Figure 4.**
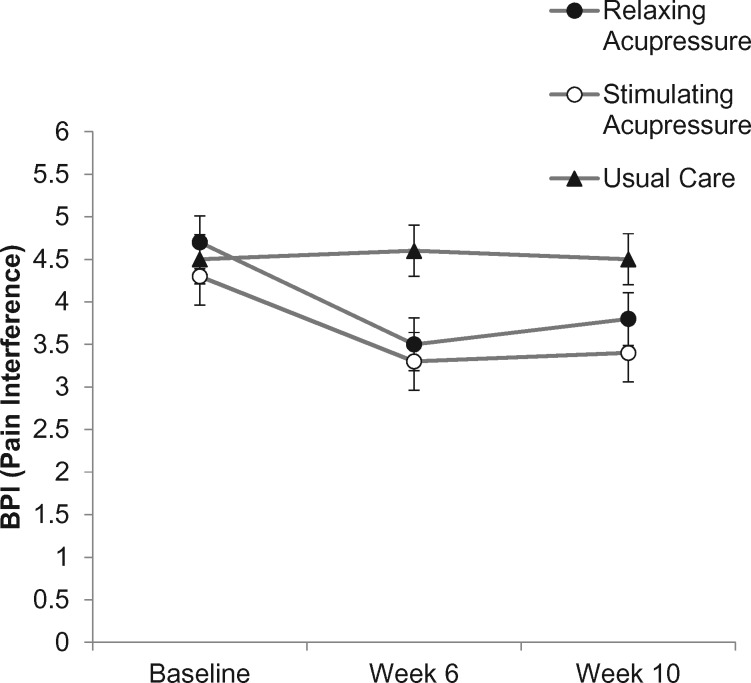
Change in pain interference across study arms (relaxing acupressure, stimulating acupressure, and usual care) as measured at baseline, 6 weeks after baseline (end of acupressure or usual care treatment), and 10 weeks after baseline (4 weeks post-acupressure or usual care treatment). BPI = Brief Pain Inventory.

At the end of treatment, stimulating acupressure was associated with statistically significantly greater reductions in pain interference than usual care (stimulating acupressure: OR = −1.07, 95% CI = −1.98 to −0.15, *P* = .017 vs usual care), but there was no statistically significant difference between relaxing acupressure and usual care (*P* = .12) or between acupressure arms (*P* > .99). Pain interference improved 25.5% and 23.2% in the stimulating and relaxing groups, respectively, but stayed the same in the usual care arm. At week 10, there were no statistically significant pairwise differences. (Table 2, Figure 4).

### Anxiety

**Figure 5.**
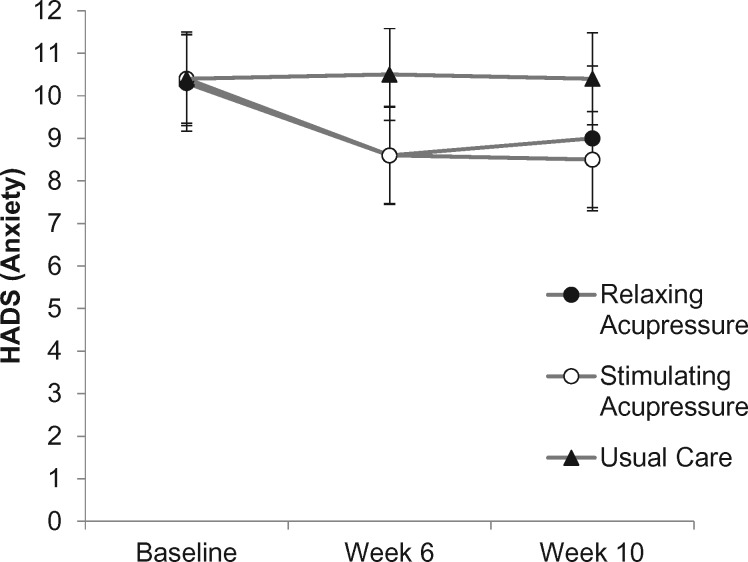
Change in anxiety across study arms (relaxing acupressure, stimulating acupressure, and usual care) as measured at baseline, 6 weeks after baseline (end of acupressure or usual care treatment), and 10 weeks after baseline (4 weeks post-acupressure or usual care treatment). HADS = Hospital Anxiety Depression Scale.

Relaxing and stimulating acupressure were associated with statistically significantly greater reductions in anxiety than usual care at the end of treatment (relaxing acupressure: OR = −1.82, 95% CI = −3.60 to −0.04, *P* = .043 vs usual care; stimulating acupressure: OR = −1.94, 95% CI = −3.66 to −0.21, *P* = .022 vs usual care), but there were no differences between the two acupressure arms (*P* = 1.000). Relaxing acupressure reduced anxiety by 16.5%, stimulating by 17.3%, with usual care showing no meaningful change. At 10 weeks, only stimulating acupressure was statistically significantly better than usual care at improving anxiety (relaxing acupressure: OR = −1.34, 95% CI = −3.17 to 0.50, *P* = .24 vs usual care; stimulating acupressure: OR = −1.99, 95% CI = −3.72 to −0.15, *P* = .02 vs usual care), while the two acupressure arms continued to show no differences (*P* = 1.000) (Table 2, Figure 5).

### Moderation and Mediation

There were no statistically significant moderators of sleep quality, anxiety, or depression. Fatigue was a statistically significant moderator (*P* = .001 overall interaction) for pain interference. Women in the relaxation acupressure group, who had less fatigue at baseline, were more likely to have lower levels of pain interference at the end of acupressure treatment compared with women in the usual care group (OR = 0.35, 95 CI = 0.15 to 0.85) (Supplementary Table 4, available online). Age was a statistically significant moderator (*P* = .032 overall interaction) for fatigue status. Women in the relaxation acupressure group who were older were more likely to have normal fatigue levels (ie, <4) at the end of treatment compared with women in the usual care group (OR = 1.1, 95% CI = 1.02 to 1.18) or in the stimulating group. Thus, for every 1-year increase in age, women were 1% more likely to respond to relaxing acupressure treatment compared with usual care or stimulating acupressure (Supplementary Table 4, available online). 

**Figure 6.**
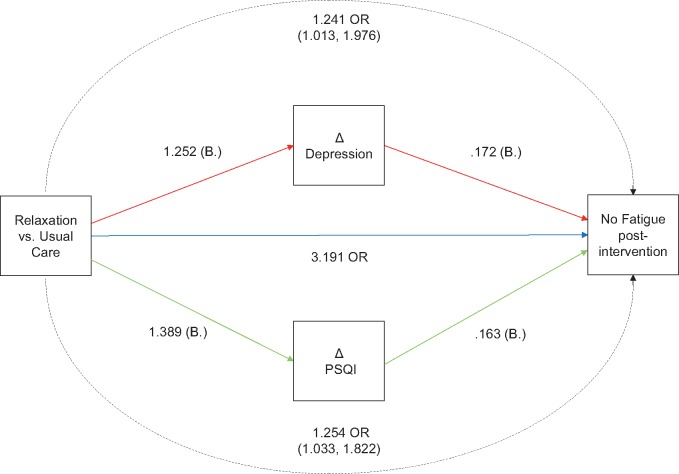
Parallel mediation model showing the direct and indirect effects of relaxing acupressure on fatigue status at week 6. All paths shown in the model are statistically significant. Covariates (age and body mass index) and non-statistically significant indirect effects are not shown. PSQI = Pittsburgh Sleep Quality Index.

There were no statistically significant mediated effects on anxiety, depression, or pain. Estimates of indirect effects are shown in Supplementary Table 5 (available online). For fatigue, there were statistically significant mediated effects of change in depressive symptoms (OR = 1.24, 95% CI = 1.01 to 1.98) and change in sleep quality (OR = 1.25, 95% CI = 1.03 to 1.82) at the end of acupressure treatment for the relaxing group vs usual care. The direct effect of relaxing acupressure was OR = 3.19, 95% CI = 1.46 to 6.97. This means that the direct effect of relaxing acupressure accounts for approximately 80% of the effect, and change in depressive symptoms and sleep quality together account for the remaining 20%. The model is shown in Figure 6.

## Discussion

Stimulating and relaxing acupressure were associated with greater improvements in anxiety than usual care; however, relaxing acupressure was associated with better reductions in depressive symptoms than stimulating acupressure (42% vs 25%), suggesting that these two types of acupressure might have different effects. There were also differences between stimulating and relaxing acupressure in how they affected pain. Only relaxing acupressure was associated with better decreases in pain severity than usual care, while only stimulating acupressure was associated with better decreases in pain interference than usual care. Although these effects were evident immediately following 6 weeks of treatment, only anxiety continued to be statistically significantly decreased for stimulating acupressure at the post-washout visit. All other symptoms were no different from usual care at post-washout, indicating that the persistence of acupressure’s effects may decrease, depending on acupressure formula and specific symptoms.

Despite improvements in most symptoms, no major factors were detected that mediated and moderated these effects. Although age was a moderator of the effect of relaxing acupressure on fatigue, this effect was mild with a decade of age having only a 10% influence on fatigue. The presence of baseline fatigue also decreased relaxing acupressure’s effect on pain interference, but this too was moderate and not observed for pain severity. No other statistically significant moderator effects were seen for multiple co-occurring symptoms. Thus, although relaxing and stimulating acupressure help multiple symptoms in BCS, the magnitude of the co-occurring factors at baseline largely had no effect on treatment outcomes. This could imply that although these symptoms co-occur, persistence and resolution of these symptoms may have distinct mechanisms from one another.

We found no mediators for the observed effects of acupressure on pain, anxiety, or depressive symptoms. Thus, although these co-occurring symptoms improved concurrently in the sample population, they did so in isolated individuals. The exception for these results was the observed effect of relaxing acupressure on fatigue. Improvements in depressive symptoms and sleep quality had a statistically significant mediation effect on decreasing fatigue. But this effect too was modest, with 80% of the relaxing acupressure’s effect being independent of improvements in sleep and depressive symptoms.

How might acupressure be working? One possibility is the placebo effect. Previous studies have shown that sham acupuncture/acupressure was also effective and had a large effect size when compared with no treatment or usual care (23,35). Extra attention received by participants because of the teaching and monitoring of the acupressure treatments may be another explanation. However, women in the acupressure groups received on average only 10 minutes of extra attention at the baseline visit compared to the usual care group. Otherwise, this study provided comparable attention in all three groups for data collection and contacts. Another explanation is the state of and plasticity of the women’s brain neurochemistry and connectivity at the time of acupressure treatment. We found, in a subset of these women, that relaxing and stimulating acupressure had different apparent effects on brain functional connectivity, suggesting the possibility that different neural mechanisms might be responsible for their different effects on different symptoms (36).

Limitations include the use of secondary data from our parent study (23). Although all participants had fatigue, not all had the other symptoms. Also, although we did detect acupressure treatment differences for depressive symptoms, we may have been underpowered for other symptoms (35). This study involved mainly white, female BCS, limiting the generalizability of our findings to other groups.

Our investigation into the effects of differing acupressure formulas for co-occurring symptoms in BCS indicates that this intervention may improve symptoms other than fatigue; however, because our findings are only hypothesis-raising, they require confirmation in an independent trial before any clinical recommendations can be made. The underlying mechanisms for these possible effects remain to be elucidated.

## Funding

This work was supported by the National Institutes of Health (grant nos. R01 CA151445 and 2UL1 TR000433-06).

## Notes

Affiliations of authors: Department of Family Medicine (SMZ) and Department of Nutritional Sciences (SMZ), University of Michigan, Ann Arbor, MI; Department of Family Medicine (ASe) and Department of Biostatistics (ASe), University of Michigan, Ann Arbor, MI; Department of Anesthesiology, University of Michigan, Ann Arbor, MI (ALH, ASc, REH); College of Nursing, Michigan State University, East Lansing, MI (GKW); Physical Medicine and Rehabilitation, University of Michigan, Ann Arbor, MI (SLM); Physical Medicine and Rehabilitation, VA Ann Arbor Health Care System, GRECC, Ann Arbor, MI (SLM); Sleep and Circadian Research Laboratory (JTA) and Departments of Psychiatry and Neurology (JTA), University of Michigan, Ann Arbor, MI.

Clinicaltrials.gov Identifier: NCT01281904.

## Supplementary Material

Supplementary Data
