# Supplementary material for: Impact of Self-Acupressure on Co-Occurring Symptoms in Cancer Survivors
Source: JNCI Cancer Spectr. 2019 Jan 16;2(4):pky064. doi: 10.1093/jncics/pky064 (PMC6334818; doi:10.1093/jncics/pky064)
Supplement: Supplementary Data [file pky064_supp.pdf]

Supplemental Table 1. Sociodemographic and Clinical Characteristics at Baseline: for Participants with Chronic Pain (Defined as Visual Analog Scale ≥ 3 at Baseline)

	Relaxing Acupressure (n=74)	Stimulating Acupressure (n=58)	Usual Care (n=61)
**Demographics**			
Age (mean years) ± SD	59.2 ± 9.3	60.8 ± 10.3	61.8 ± 9.3
Race n (%)			
White	67 (91)	50 (86)	51 (84)
**Clinical Characteristics**			
BFI (mean ± SD)[1]	5.2 ± 1.5	5.4 ± 1.5	5.2 ± 1.4
BPI (mean ± SD)[1]			
Severity	4.8 ± 1.7	4.6 ± 1.8	4.4 ± 1.9
Interference	3.9 ± 2.2	4.1 ± 2.3	3.9 ± 2.3
HADS (mean ± SD)[1]			
Anxiety	8.0 ± 4.2	8.7 ± 3.5	8.0 ± 3.9
Depression	6.1 ± 3.0	6.9 ± 3.2	6.0 ± 3.4
Stage of Cancer n (%)			
Stage 0[2]	15 (20)	9 (16)	19 (31)
Stage 1	22 (30)	21 (36)	21 (34)
Stage 2	21 (28)	19 (33)	8 (13)
Stage 3	11 (15)	4 (7)	7 (12)
Unknown	4 (5)	4 (7)	6 (10)
Estrogen Receptor Status			
Yes	47 (64)	44 (76)	38 (62)
No	16 (22)	6 (10)	13 (21)
Unknown	11 (15)	8 (14)	10 (16)
Menopausal Status			
Premenopausal	25 (34)	20 (35)	21 (34)

Perimenopausal	7 (10)	5 (9)	4 (7)
Postmenopausal	40 (54)	33 (57)	32 (53)
Unknown	2 (3)	0 (0)	4 (7)
Time Since Cancer Diagnosis in years (mean ± SD) [3]	5.1 ± 2.0	5.6 ± 3.1	6.3 ± 5.7
Treatments (were received) n (%)[4]			
Surgery	74 (100)	58 (100)	60 (98)
Chemotherapy	39 (53)	26 (45)	20 (33)
Radiation	54 (73)	43 (74)	44 (72)
Hormone Therapy	46 (62)	46 (79)	35 (58)
Medications (% taking at baseline)			
Acetaminophen	26 (36)	16 (28)	11 (19)
Anticonvulsants	11 (15)	9 (16)	8 (14)
Aromatase Inhibitors	13 (18)	16 (28)	15 (26)
Benzodiazepines	10 (14)	5 (9)	7 (12)
Glucosamine/Chondroitin	7 (10)	3 (5)	10 (17)
NDRIs[5]	2 (3)	3 (5)	6 (10)
NSAIDs[5]	48 (67)	37 (65)	39 (67)
Omega 3s	15 (21)	24 (42)	20 (35)
Opiates & Morphinomimetrics	18 (25)	9 (16)	7 (12)
SERMs[5]	8 (11)	10 (18)	6 (10)
SNRIs[5]	11 (15)	10 (18)	8 (14)
SSRIs[5]	16 (22)	11 (19)	11 (19)
Tricyclics	3 (4)	2 (4)	3 (5)
Other[6]	7 (10)	5 (9)	9 (16)

[1] BFI=Brief Fatigue Inventory; BPI=Brief Pain Inventory; HADS=Hospital Anxiety and Depression Scale
[2] Stage 0 includes ductal carcinoma in situ (DCIS) and Lobular carcinoma in situ (LCIS)
[3] Time since Cancer Diagnosis" was calculated from on-study date and date of diagnosis in years
[4] Percentages may not add up to 100% because participants can receive multiple treatments or diagnoses
[5] NDRIs=Norepinephrine-Dopamine Reuptake Inhibitors; NSAIDs=Nonsteroidal Anti-Inflammatory Drugs; SERMs=Selective Estrogen Receptor Modulators; SNRIs=Serotonin-Norepinephrine Reuptake Inhibitors; SSRIs=Selective Serotonin Reuptake Inhibitors
[6] Other medications include Trazodone, Lisinopril, Flexeril, Fioricet, Imitrex, Norgesic, Fiorinal, Butalbital, Remeron, Buspirone, Milk Thistle, Ginger Biloba, Medical marijuana, St. John's Wort, Pramipexole, Requip, Adderall, Buspar, Tamoxilen, Atenolol, Omeprazole, Baby Aspirin, Vitamin D, Vitamin E, and topical Lidocaine

Supplemental Table 2. Sociodemographic and Clinical Characteristics at Baseline for Participants with Baseline Anxiety (Defined as Hospital Anxiety Depression Sub Scale, Anxiety ≥ 8 at Baseline)

	Relaxing Acupressure (n= 42)	Stimulating Acupressure (n= 56)	Usual Care (n= 44)
**Demographics**			
Age (mean years) ± SD	59.0 ± 9.4	59.9 ± 9.2	58.6 ± 9.7
Race n (%)	38 (91)	47 (84)	36 (82)
White			
**Clinical Characteristics**			
BFI (mean ± SD)[1]	5.4 ± 1.6	5.3 ± 1.6	5.1 ± 1.6
BPI (mean ± SD)[1]			
Severity	4.6 ± 2.1	3.9 ± 2.2	3.9 ± 1.9
Interference	4.1 ± 2.6	3.5 ± 2.2	3.6 ± 2.3
HADS (mean ± SD)[1]			
Anxiety	11.3 ± 2.9	10.7 ± 2.3	11.2 ± 2.9
Depression	7.1 ± 3.0	8.1 ± 3.0	7.4 ± 3.0
Stage of Cancer n (%)			
Stage 0[2]	9 (21)	12 (22)	17 (39)
Stage 1	14 (33)	20 (36)	11 (25)
Stage 2	9 (21)	15 (27)	8 (18)
Stage 3	6 (14)	4 (7)	4 (9)
Unknown	3 (7)	4 (7)	4 (9)
Estrogen Receptor Status			
Yes	25 (60)	37 (66)	28 (64)
No	9 (21)	10 (18)	9 (21)
Unknown	8 (19)	9 (16)	7 (16)
Menopausal Status			
Premenopausal	13 (31)	20 (36)	22 (50)

Perimenopausal	6 (14)	5 (9)	2 (5)
Postmenopausal	21 (50)	31 (55)	18 (41)
Unknown	2 (5)	0 (0)	2 (5)
Time Since Cancer Diagnosis in years (mean ± SD)[3]	5.3 ± 2.6	5.4 ± 2.7	5.2 ± 4.5
Treatments (were received) n (%)[4]			
Surgery	42 (100)	56 (100)	43 (98)
Chemotherapy	22 (52)	23 (41)	15 (34)
Radiation	31 (74)	39 (70)	28 (64)
Hormone Therapy	23 (55)	40 (71)	26 (61)
Medications (% taking at baseline)			
Acetaminophen	12 (30)	13 (24)	9 (21)
Anticonvulsants	7 (18)	6 (11)	6 (14)
Aromatase Inhibitors	5 (13)	15 (28)	11 (26)
Benzodiazepines	9 (23)	6 (11)	8 (19)
Glucosamine/Chondroitin	5 (13)	3 (6)	6 (14)
NDRIs[5]	2 (5)	6 (11)	3 (7)
NSAIDs[5]	28 (70)	34 (63)	30 (71)
Omega 3s	3 (8)	20 (37)	12 (29)
Opiates & Morphinomimetrics	11 (28)	8 (15)	2 (5)
SERMs[5]	6 (15)	10 (19)	6 (14)
SNRIs[5]	6 (15)	12 (22)	6 (14)
SSRIs[5]	8 (20)	10 (19)	12 (29)
Tricyclics	1 (3)	2 (4)	3 (7)
Other[6]	4 (10)	6 (11)	5 (12)

[1] BFI=Brief Fatigue Inventory; BPI=Brief Pain Inventory; HADS=Hospital Anxiety and Depression Scale
[2] Stage 0 includes ductal carcinoma in situ (DCIS) and Lobular carcinoma in situ (LCIS)
[3] Time since Cancer Diagnosis" was calculated from on-study date and date of diagnosis in years
[4] Percentages may not add up to 100% because participants can receive multiple treatments or diagnoses
[5] NDRIs=Norepinephrine-Dopamine Reuptake Inhibitors; NSAIDs=Nonsteroidal Anti-Inflammatory Drugs; SERMs=Selective Estrogen Receptor Modulators; SNRIs=Serotonin-Norepinephrine Reuptake Inhibitors; SSRIs=Selective Serotonin Reuptake Inhibitors
[6] Other medications include Trazodone, Lisinopril, Flexeril, Fioricet, Imitrex, Norgesic, Fiorinal, Butalbital, Remeron, Buspirone, Milk Thistle, Ginger Biloba, Medical marijuana, St. John's Wort, Pramipexole, Requip, Adderall, Buspar, Tamoxilen, Atenolol, Omeprazole, Baby Aspirin, Vitamin D, Vitamin E, and topical Lidocaine

Supplemental Table 3. Sociodemographic and Clinical Characteristics at Baseline for Participants with Baseline Depression (Defined as Hospital Anxiety Depression Sub Scale, Depression ≥ 8 at Baseline)

	Relaxing Acupressure (n=27)	Stimulating Acupressure (n=36)	Usual Care (n=29)
**Demographics**			
Age (mean years) ± SD	58.0 ± 9.9	62.2 ± 8.6	59.8 ± 7.8
Race n (%)	25 (93)	29 (81)	23 (79)
White			
**Clinical Characteristics**			
BFI (mean ± SD)[1]	5.4 ± 1.7	5.8 ± 1.5	5.6 ± 1.4
BPI (mean ± SD)[1]			
Severity	4.6 ± 2.2	3.7 ± 2.6	4.3 ± 1.9
Interference	4.3 ± 2.5	3.5 ± 2.5	4.4 ± 2.4
HADS (mean ± SD)[1]			
Anxiety	9.6 ± 3.8	10.5 ± 3.1	10.2 ± 4.1
Depression	9.8 ± 1.8	10.1 ± 1.8	9.9 ± 1.8
Stage of Cancer n (%)			
Stage 0[2]	4 (15)	8 (22)	7 (24)
Stage 1	12 (44)	14 (39)	8 (28)
Stage 2	7 (26)	8 (22)	5 (17)
Stage 3	2 (7)	2 (6)	6 (21)
Unknown	2 (7)	3 (8)	3 (10)
Estrogen Receptor Status			
Yes	16 (59)	23 (64)	20 (69)
No	6 (22)	6 (17)	6 (21)
Unknown	5 (19)	7 (19)	3 (10)
Menopausal Status			
Premenopausal	7 (26)	9 (25)	13 (45)

Perimenopausal	3 (11)	3 (8)	4 (14)
Postmenopausal	16 (59)	24 (67)	10 (35)
Unknown	1 (4)	0 (0)	2 (7)
Time Since Cancer Diagnosis in years (mean ± SD) [3]	5.4 ± 2.3	4.9 ± 1.8	5.5 ± 3.5
Treatments (were received) n (%)[4]			
Surgery	27 (100)	36 (100)	28 (97)
Chemotherapy	12 (44)	14 (39)	13 (45)
Radiation	18 (67)	27 (75)	18 (62)
Hormone Therapy	17 (63)	25 (69)	19 (66)
Medications (% taking at baseline)			
Acetaminophen	10 (37)	11 (31)	8 (29)
Anticonvulsants	2 (7)	5 (14)	4 (14)
Aromatase Inhibitors	4 (15)	7 (20)	8 (29)
Benzodiazepines	2 (7)	4 (11)	8 (29)
Glucosamine/Chondroitin	4 (15)	2 (6)	2 (7)
NDRIs[5]	0 (0)	4 (11)	3 (11)
NSAIDs[5]	18 (67)	20 (57)	15 (54)
Omega 3s	7 (26)	14 (40)	7 (25)
Opiates & Morphinomimetrics	8 (30)	5 (14)	2 (7)
SERMs[5]	4 (15)	8 (23)	6 (21)
SNRIs[5]	4 (15)	10 (29)	6 (21)
SSRIs[5]	4 (15)	9 (26)	9 (32)
Tricyclics	1 (4)	2 (6)	2 (7)
Other[6]	4 (15)	4 (11)	2 (7)

[1] BFI=Brief Fatigue Inventory; BPI=Brief Pain Inventory; HADS=Hospital Anxiety and Depression Scale

[2] Stage 0 includes ductal carcinoma in situ (DCIS) and Lobular carcinoma in situ (LCIS)

[3] Time since Cancer Diagnosis" was calculated from on-study date and date of diagnosis in years

[4] Percentages may not add up to 100% because participants can receive multiple treatments or diagnoses

[5] NDRIs=Norepinephrine-Dopamine Reuptake Inhibitors; NSAIDs=Nonsteroidal Anti-Inflammatory Drugs; SERMs=Selective Estrogen Receptor Modulators; SNRIs=Serotonin-Norepinephrine Reuptake Inhibitors; SSRIs=Selective Serotonin Reuptake Inhibitors

[6] Other medications include Trazodone, Lisinopril, Flexeril, Fioricet, Imitrex, Norgesic, Fiorinal, Butalbital, Remeron, Buspirone, Milk Thistle, Ginger Biloba, Medical marijuana, St. John's Wort, Pramipexole, Requip, Adderall, Buspar, Tamoxilen, Atenolol, Omeprazole, Baby Aspirin, Vitamin D, Vitamin E, and topical Lidocaine

Supplemental Table 4. Impact of Potential Moderators on Response Self-Acupressure to Patient Reported Outcomes At End of Acupressure Treatment

Patient Reported Outcome	Moderator	RA[2] vs UC[2]			SA[2] vs UC[2]			SA[2] vs RA[2]		
		Odds Ratio[3]	LLCI[2]	ULCI[2]	Odds Ratio[3]	LLCI[2]	ULCI	Odds Ratio[3]	LLCI[2]	ULCI[2]
**Depressive Mood[1]**										
	Anxiety[1]	1.04	0.67	1.63	0.90	0.62	1.31	0.87	0.56	1.35
	Fatigue[1]	0.63	0.22	1.78	0.85	0.32	2.29	1.36	0.52	3.56
	Pain Interference[1]	1.13	0.56	2.26	0.91	0.54	1.53	0.81	0.42	1.55
	Sleep Quality[1]	0.80	0.51	1.24	0.83	0.56	1.21	1.04	0.71	1.52
	Age	0.93	0.78	1.11	0.99	0.85	1.14	1.06	0.89	1.26
	BMI[2] kg/m^2	0.89	0.66	1.20	0.79	0.62	1.01	0.89	0.66	1.21
**Anxiety[1]**										
	Depression[1]	1.02	0.67	1.55	1.08	0.74	1.59	1.06	0.76	1.48
	Fatigue[1]	1.22	0.55	2.71	1.00	0.48	2.07	0.82	0.39	1.71
	Pain Interference[1]	1.27	0.77	2.09	1.22	0.76	1.96	0.96	0.61	1.50
	Sleep Quality[1]	1.14	0.83	1.56	0.97	0.73	1.30	0.85	0.65	1.12
	Age	0.93	0.82	1.05	0.94	0.83	1.06	1.02	0.91	1.14
	BMI[2] $kg/m^{2,}$	1.04	0.86	1.26	1.02	0.86	1.21	0.98	0.83	1.17
**Sleep Quality[1]**										
	Anxiety[1]	0.88	0.61	1.26	0.87	0.60	1.27	1.00	0.76	1.30
	Depression[1]	0.67	0.41	1.09	0.70	0.43	1.14	1.04	0.77	1.39
	Fatigue[1]	0.58	0.25	1.35	0.50	0.21	1.19	0.86	0.42	1.74
	Pain Interference[1]	0.77	0.43	1.39	0.75	0.42	1.33	0.97	0.67	1.41
	Age	1.03	0.90	1.17	1.03	0.90	1.17	1.00	0.91	1.11
	BMI[2] kg/m^2	0.92	0.72	1.16	0.91	0.72	1.14	0.99	0.84	1.16
**Pain Interference[1]**										
	Anxiety[1]	1.01	0.82	1.26	1.17	0.91	1.50	1.14	0.89	1.46
	Depression[1]	0.77	0.57	1.02	0.86	0.64	1.15	1.09	0.84	1.43
	Fatigue[1]	0.35	0.15	0.85	0.46	0.18	1.19	1.36	0.69	2.71
	Sleep Quality[1]	0.88	0.71	1.11	0.87	0.68	1.12	0.99	0.79	1.23
	Age	1.11	1.01	1.22	1.06	0.96	1.17	0.95	0.87	1.03
	BMI[2] $kg/m^{2,}$	0.92	0.78	1.07	0.92	0.80	1.07	1.01	0.88	1.16
**Fatigue[1]**										
	Anxiety[1]	0.93	0.78	1.11	1.01	0.84	1.21	1.09	0.90	1.33
	Depression[1]	0.81	0.64	1.02	0.85	0.68	1.06	1.05	0.84	1.32
	Pain Interference[1]	0.77	0.55	1.08	0.83	0.59	1.16	1.08	0.80	1.46
	Sleep Quality[1]	0.89	0.74	1.07	0.80	0.65	0.99	0.91	0.74	1.10
	Age	1.1	1.02	1.18	1.01	0.94	1.08	0.92	0.86	0.98
	BMI[2] kg/m^2	0.99	0.88	1.12	0.95	0.85	1.06	0.96	0.85	1.08

1. Patient Reported Outcomes Derived From: Anxiety = Hospital Anxiety Depression Scale Anxiety Subscale; Depression = Hospital Anxiety Depression Scale Depression Subscale; Fatigue = Brief Fatigue Inventory; Pain Interference = Brief Pain Inventory Interference Subscale; and Sleep Quality = Pittsburgh Sleep Quality Global Score.
2. UC = Usual Care Group; RA = Relaxing Acupressure Group; SA = Stimulating Acupressure Group; LLCI = Lower Limit Confidence Interval; and ULCI = Upper Limit Confidence Interval; BMI = Body Mass Index.
3. Odds Ratio and Bias Corrected 95 % Confidence Intervals derived from Logistic Regression
Bolded entries are statistically significant $p<0.05$

Supplemental Table 5. Impact of Potential Mediators of Self-Acupressure on Response to Patient Reported Outcomes At End of Acupressure Treatment1										
**Patient Reported Outcome**	**Mediator**	**UC[2] vs RA[2]**			**UC[2] vs SA[2]**			**RA[2] vs SA[2]**		
		**Estimate[3]**	**LLCI[2]**	**ULCI[2]**	**Estimate[3]**	**LLCI[2]**	**ULCI**	**Estimate[3]**	**LLCI[2]**	**ULCI[2]**
**Depressive Mood[1]**										
	Δ Anxiety[2]	1.08	0.12	7918.05	0.98	0.08	2.48	1.00	0.04	10.70
	Δ Fatigue[2]	0.73	0.00	61.65	0.61	0.00	1.95	1.04	0.00	5123.02
	Δ Pain Interference[2]	1.11	0.14	12315.33	1.08	0.48	7.97	0.82	0.01	261.81
	Δ Sleep Quality[2]	1.06	0.09	88.68	1.06	0.71	19.79	1.76	0.05	69.24
**Anxiety[1]**										
	Δ Depressive Mood[2]	0.86	0.06	1.45	0.87	0.27	3.19	0.98	0.49	1.55
	Δ Fatigue[2]	1.03	0.18	9.44	0.78	0.18	2.95	1.00	0.67	1.45
	Δ Pain Interference[2]	0.95	0.15	1.43	0.99	0.51	1.35	1.00	0.64	1.27
	Δ Sleep Quality[2]	0.88	0.19	1.56	0.94	0.39	1.21	0.97	0.53	1.29
**Sleep Quality[1]**										
	Δ Anxiety[2]	0.99	0.77	1.10	1.03	0.91	1.49	0.99	0.79	1.08
	Δ Depressive Mood[2]	0.89	0.59	1.09	0.90	0.62	1.35	1.00	0.80	1.16
	Δ Fatigue[2]	0.90	0.56	1.18	0.78	0.46	1.06	1.02	0.69	1.51
	Δ Pain Interference[2]	0.87	0.50	1.04	0.95	0.68	1.13	1.00	0.85	1.11
**Pain Interference[1]**										
	Δ Anxiety[2]	1.01	0.90	1.36	0.99	0.70	1.23	0.98	0.68	1.13
	Δ Depressive Mood[2]	0.95	0.60	1.27	1.08	0.50	1.96	1.02	0.89	1.62
	Δ Fatigue[2]	0.88	0.51	1.08	0.96	0.60	1.54	1.04	0.88	1.54
	Δ Sleep Quality[2]	0.94	0.58	1.30	0.88	0.48	1.06	0.97	0.65	1.11
**Fatigue[1]**										
	Δ Anxiety[2]	1.02	0.97	1.25	0.90	0.67	1.01	0.97	0.77	1.05
	Δ Depressive Mood[2]	0.80	0.52	0.97	0.83	0.57	1.01	1.04	0.77	1.48
	Δ Pain Interference[2]	0.89	0.67	1.00	0.94	0.73	1.05	1.00	0.91	1.08
	Δ Sleep Quality[2]	0.80	0.58	0.96	0.87	0.64	1.00	0.96	0.78	1.08

1. The table shows the estimate of mediation effect along with the associated confidence interval. An effect estimate significantly different from 1 indicates mediation
2. Patient Reported Outcomes Derived From: Anxiety = Hospital Anxiety Depression Scale Anxiety Subscale; Depression = Hospital Anxiety Depression Scale Depression Subscale; Fatigue = Brief Fatigue Inventory; Pain Interference = Brief Pain Inventory Interference Subscale; and Sleep Quality = Pittsburgh Sleep Quality Global Score.
2. UC = Usual Care Group; RA = Relaxing Acupressure Group; SA = Stimulating Acupressure Group; LLCI = Lower Limit Confidence Interval; and ULCI = Upper Limit Confidence Interval.
3. Estimates and Bias Corrected 95 % Bootstrapped Confidence Intervals (20,000 samples) derived from Logistic Regression using PROCESS Command SPSS

Relaxation Acupressure Points

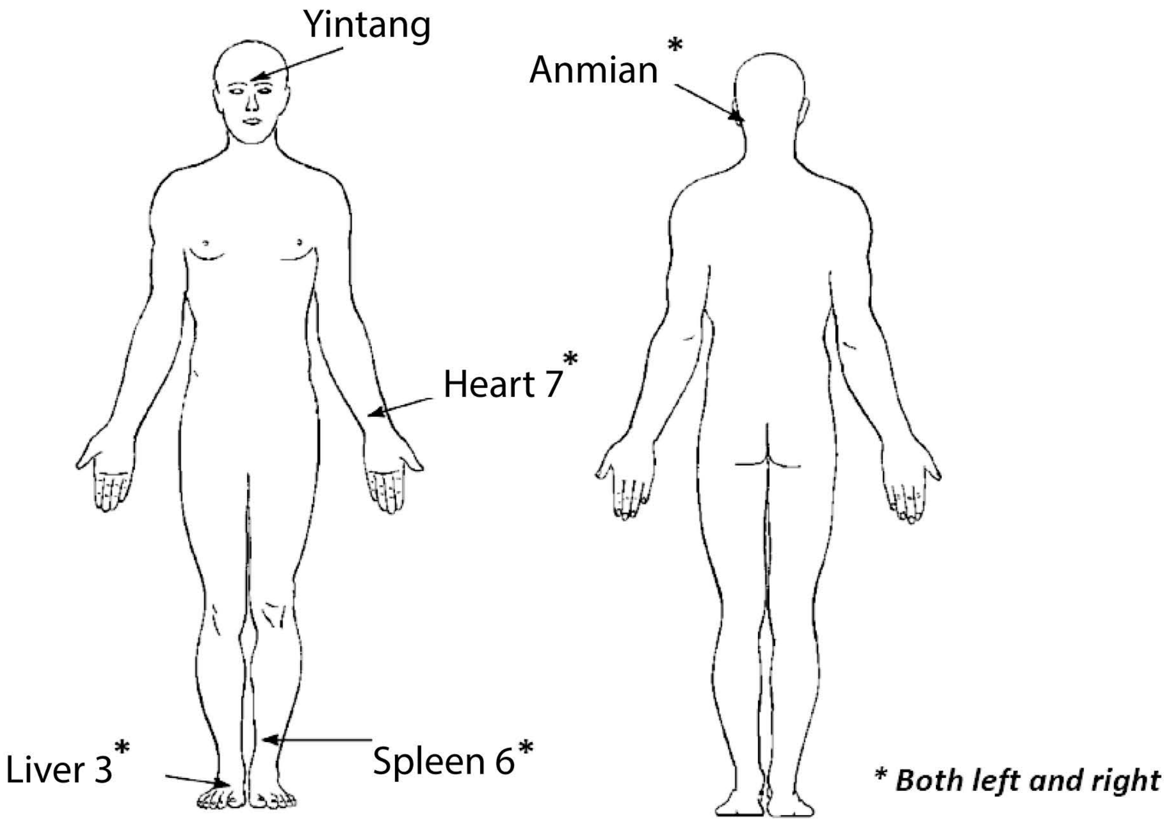

Stimulation Acupressure Points

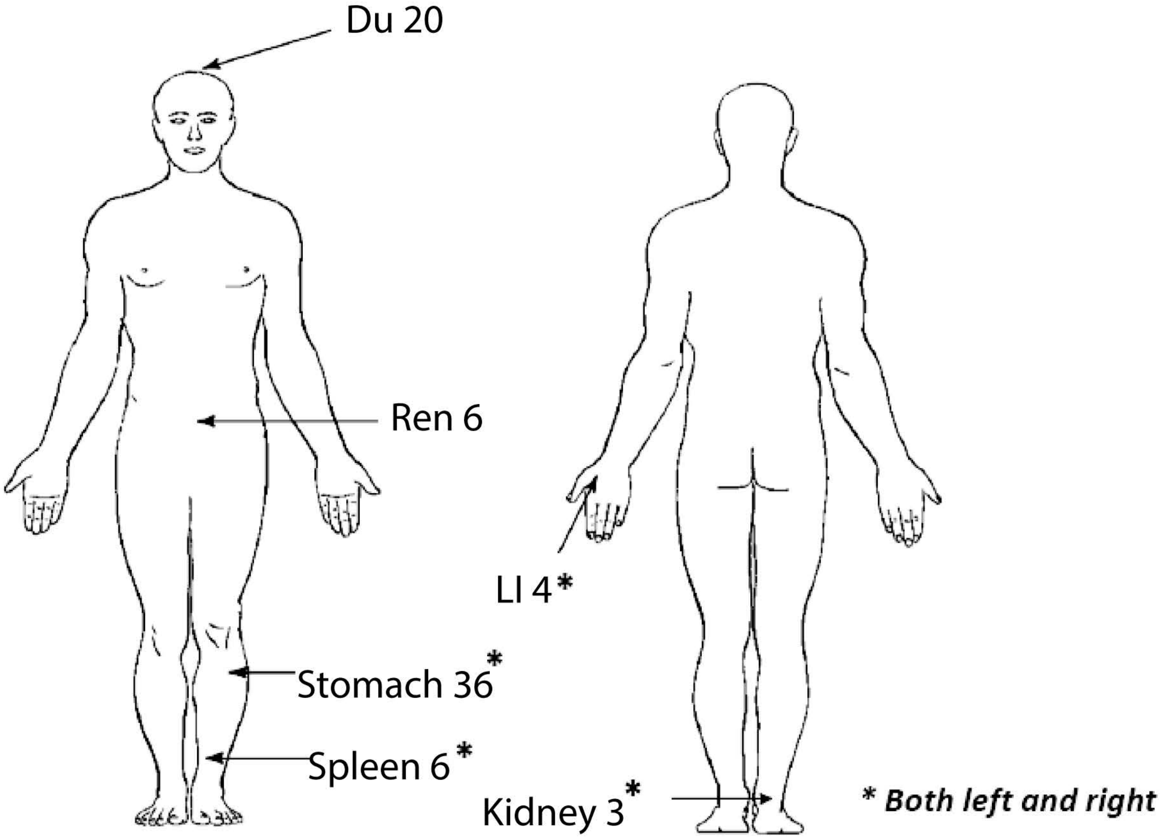

Supplemental Figure 2

Consort Flow Diagram for Participants with Chronic Pain at Baseline (defined as VAS≥3)

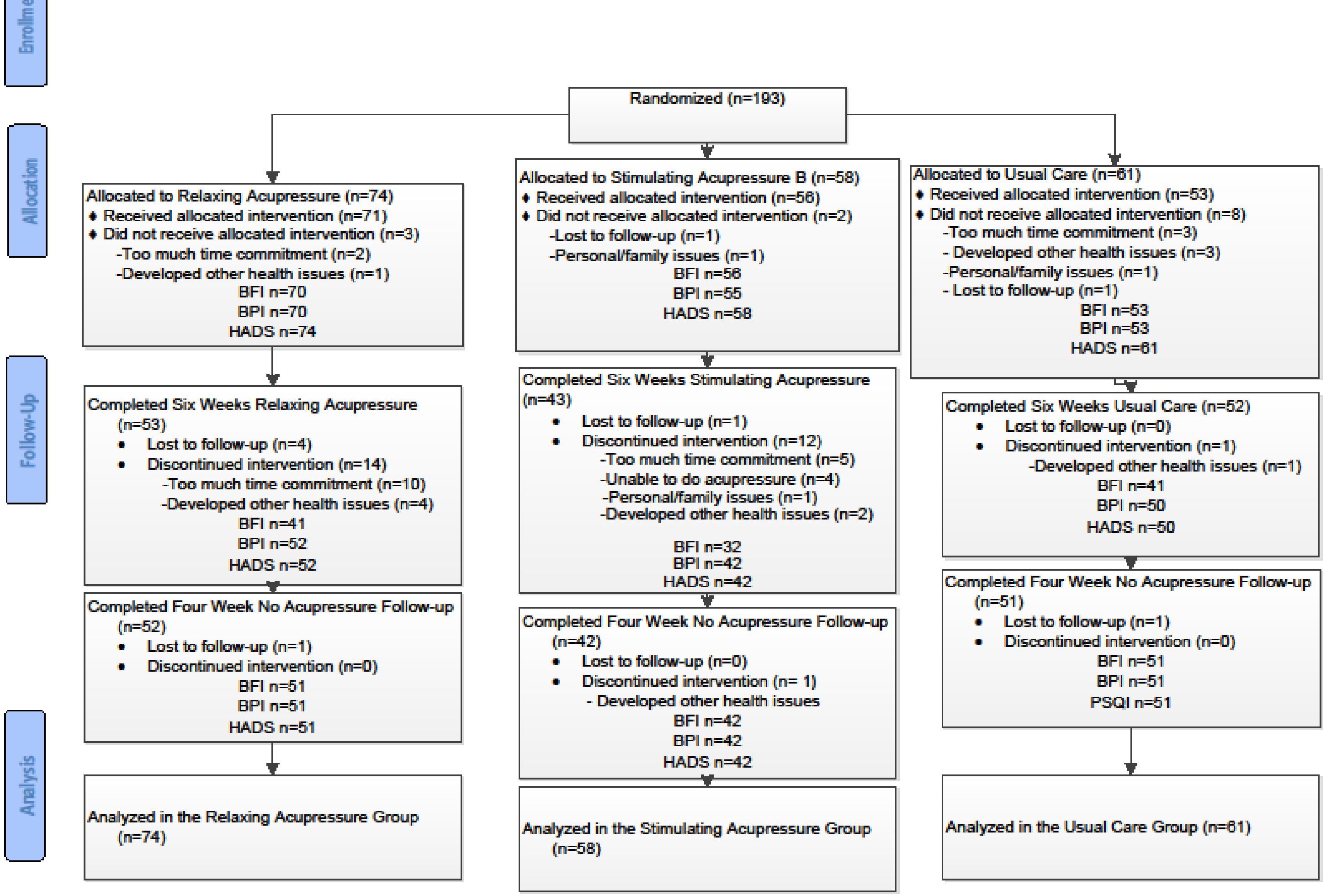

Supplemental Figure 3

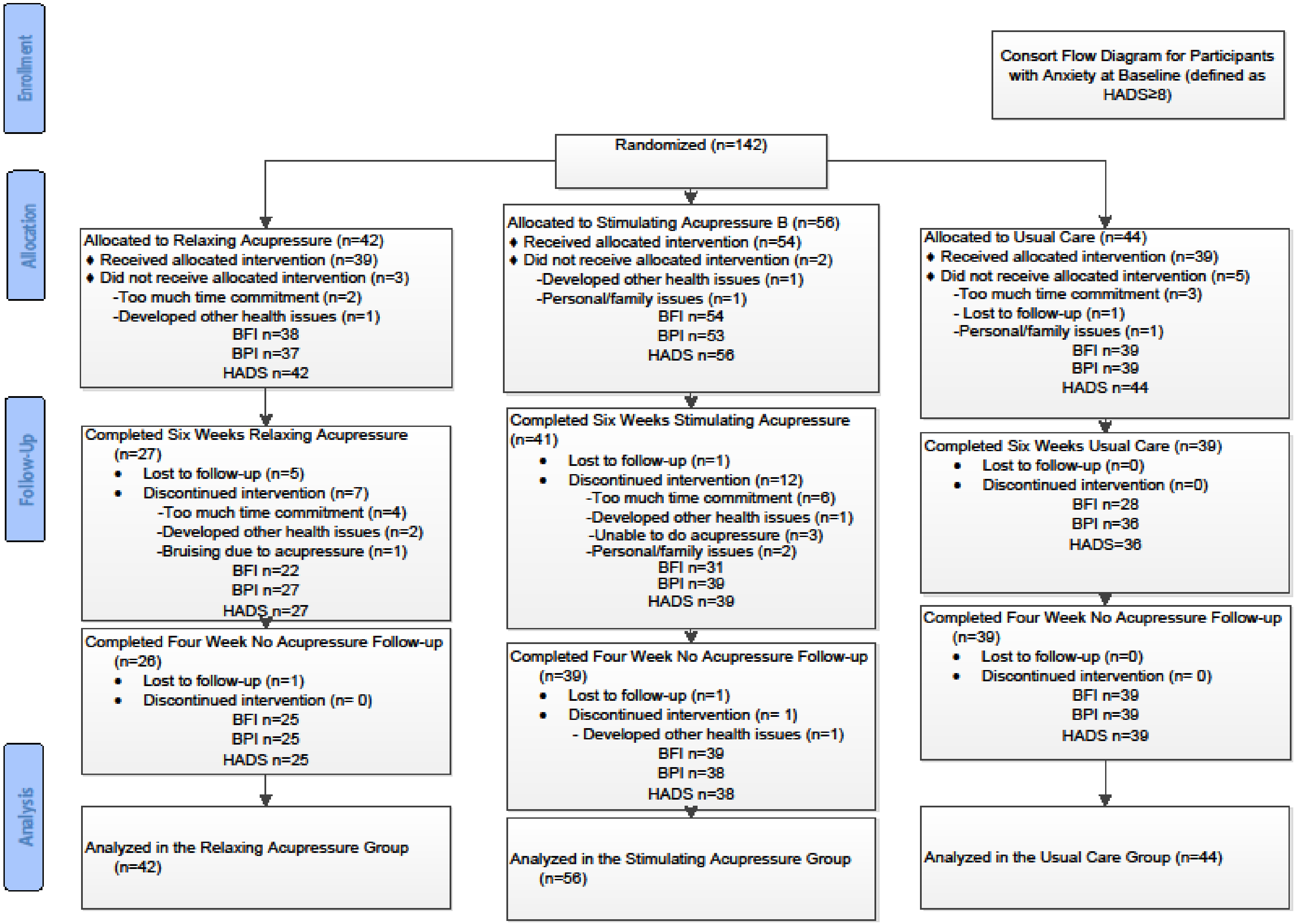

Supplemental Figure 4

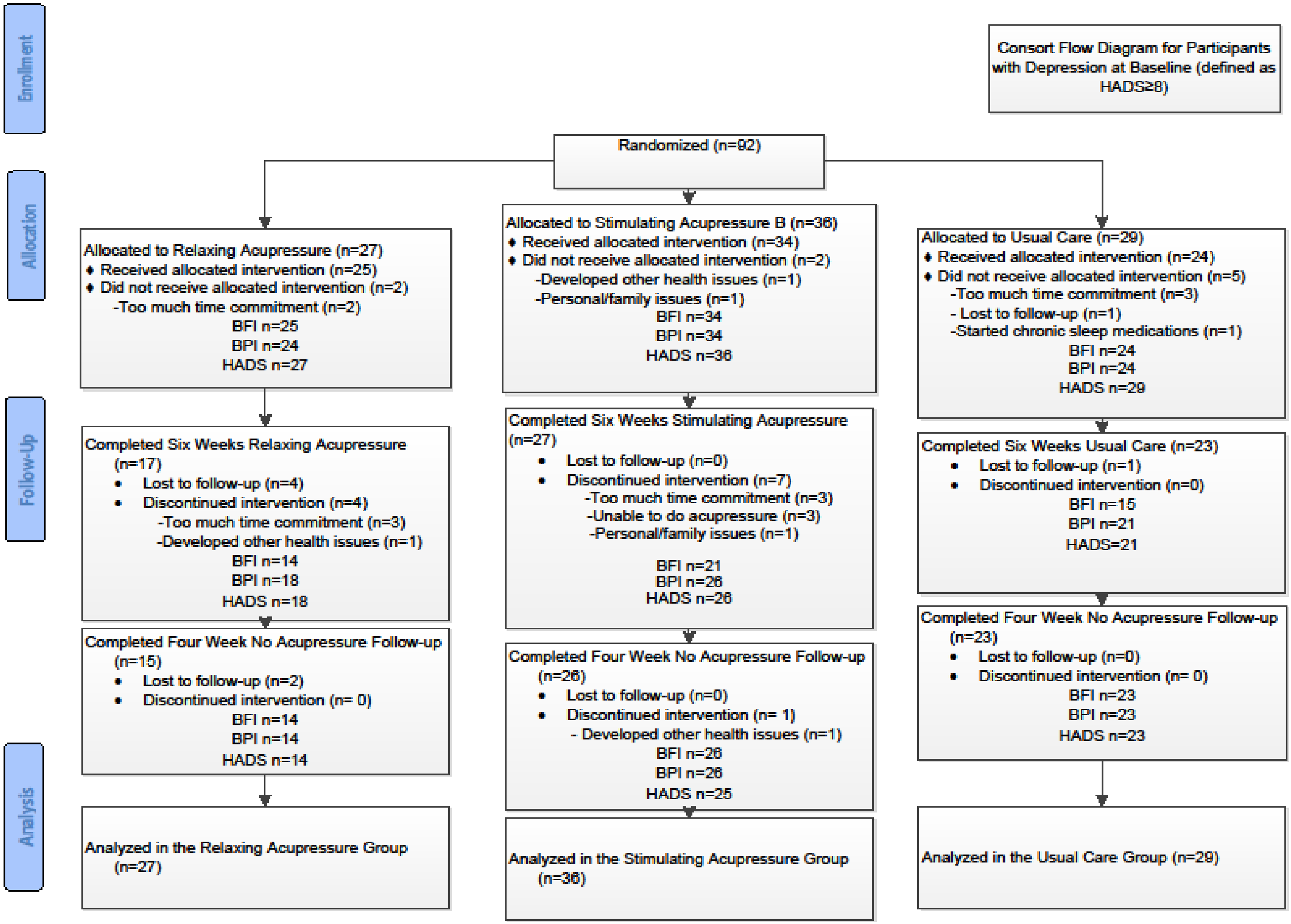

Supplemental Figure Legend

Supplemental Figure 1. Location of relaxing and stimulating self-acupressure points. Points designated with an asterisk were performed on both the left and right hand sides of the body.

Supplemental Figure 2. Consort Flow Diagram for Participants with Chronic Pain at Baseline (Defined as Visual Analog Scale $\geq$3).

Supplemental Figure 3. Consort Flow Diagram for Participants with Anxiety at Baseline (defined as Hospital Anxiety Depression Anxiety Sub score, Defined as Score $\geq$8).

Supplemental Figure 4. Consort Flow Diagram for Participants with Depression at Baseline (defined as Hospital Anxiety Depression Sub score, Defined as Score $\geq$8).
